# Severe Pediatric Arterial Thrombosis in Antiphospholipid Syndrome and Systemic Lupus Erythematosus: A Case Report

**DOI:** 10.7759/cureus.91824

**Published:** 2025-09-08

**Authors:** Isabela Jimenez, Hendrick Trelles Sanchez, Scarleth Samantha Gómez, Heidi Zuñiga González, Mishell Estefanía Llerena Vargas, Dajhana Gleidy Di Valverde Bonilla, Carlos Valladares

**Affiliations:** 1 General Medicine, University of Manizales, Manizales, COL; 2 Pediatric Rheumatology, Catholic University of Santa María, Arequipa, PER; 3 Pediatric Rheumatology, Catholic University of Honduras, Tegucigalpa, HND; 4 General Medicine, University of Monterrey, Monterrey, MEX; 5 General Medicine, Equinox University of Technology, Quito, ECU; 6 Pediatric Rheumatology, Aquino University Bolivia, Santa Cruz, BOL; 7 Internal Medicine, Robert Wood Johnson (RWJ) Barnabas Health, Toms River, USA

**Keywords:** adolescents, antiphospholipid syndrome, autoimmune disorders, case report, pediatric rheumatology, systemic lupus erythematosus, thrombosis, vascular occlusion

## Abstract

Systemic lupus erythematosus (SLE) is a chronic, multisystem immune-mediated disease that can affect any organ system. One of its most significant complications is antiphospholipid syndrome (APS), an autoimmune disorder characterized by thrombosis, which typically presents with venous thrombosis in children. We report a 14-year-old previously healthy girl presenting with progressive right foot cyanosis and ulcerative lesions. Examination revealed digital necrosis, absent distal pulses, and delayed capillary refill. Laboratory findings included elevated antiphospholipid antibodies (aPLs), positive antinuclear antibodies (ANAs), hypocomplementemia, prolonged activated partial thromboplastin time (aPTT), and a reactive Venereal Disease Research Laboratory (VDRL) test. Imaging confirmed occlusion of the anterior tibial and peroneal arteries. She was diagnosed with critical limb ischemia due to APS secondary to SLE. Treatment with intravenous methylprednisolone, anticoagulation, antiplatelet agents, and vasoprotective therapy led to significant improvement, avoiding surgical intervention. APS secondary to SLE is uncommon in pediatric patients, and arterial thrombosis is an especially rare initial manifestation. Juvenile SLE often presents with non-specific symptoms such as arthralgias, headache, fever, and weight loss, which were absent in our patient. Instead, initial symptoms included intermittent claudication-like pain and digital necrosis, likely resulting from endothelial cell proliferation, intimal hyperplasia, endothelial dysfunction, platelet activation, and complement consumption, leading to progressive luminal narrowing and thrombosis. The lack of validated pediatric classification criteria for APS secondary to SLE complicates management decisions. This case underscores the importance of early recognition and prompt initiation of anticoagulation, immunosuppression, and vasoprotective therapy to prevent irreversible tissue damage and the need for surgical intervention.

## Introduction

Systemic lupus erythematosus (SLE) is a chronic, multisystem immune-mediated disease that can affect any organ system, such as the skin, joints, kidneys, and cardiovascular system. This condition predominantly affects adults; however, it may manifest before the age of 16 years in 10-20% of all cases, a condition referred to as juvenile-onset SLE. It often presents with more severe manifestations, a high prevalence of pre-existing organ damage at diagnosis, more complications, and less favorable outcomes compared to adult-onset SLE [1]. One of the most significant complications associated with SLE is antiphospholipid syndrome (APS), an autoimmune disease characterized by arterial, venous, or microvascular thrombosis. It can be classified as primary when there is no underlying disorder, or as secondary when it is associated with another disease, most commonly SLE [2]. The most common complication in children with APS is venous thrombosis [3]; therefore, presenting arterial thrombosis with digital necrosis is atypical and may represent the initial and rare manifestation of APS secondary to SLE [4]. There are a few reports documenting the coexistence of SLE and APS in pediatric patients, highlighting the importance of early recognition and management to avoid severe complications.

In APS, antiphospholipid antibodies (aPLs) are directed mainly against phospholipid-binding proteins, such as β2-glycoprotein I and prothrombin, leading to activation of endothelial cells, platelets, and the complement system. This results in a procoagulant and proinflammatory state that promotes acute thrombosis and chronic vasculopathy. In patients with SLE, chronic inflammation and endothelial injury may further stimulate persistent aPL production, increasing thrombotic risk, particularly in those with lupus anticoagulant positivity, which is the strongest predictor of thrombosis in pediatric SLE [2].

In this case report, we present a 14-year-old patient with arterial thrombosis and digital necrosis as the initial manifestation of APS secondary to SLE, whose successful management led to the avoidance of limb amputation.

## Case presentation

A 14-year-old girl with no prior medical history presented to a tertiary pediatric hospital with a four-month history of cyanosis in the fourth and fifth toes of her right foot, triggered by exercise, and associated with intermittent claudication-type pain. Her symptoms worsened over the two weeks preceding admission, progressing to ulcerative lesions on the hallux and fourth and fifth toes of the right foot with serous discharge and distal coldness.

**Figure 1:**
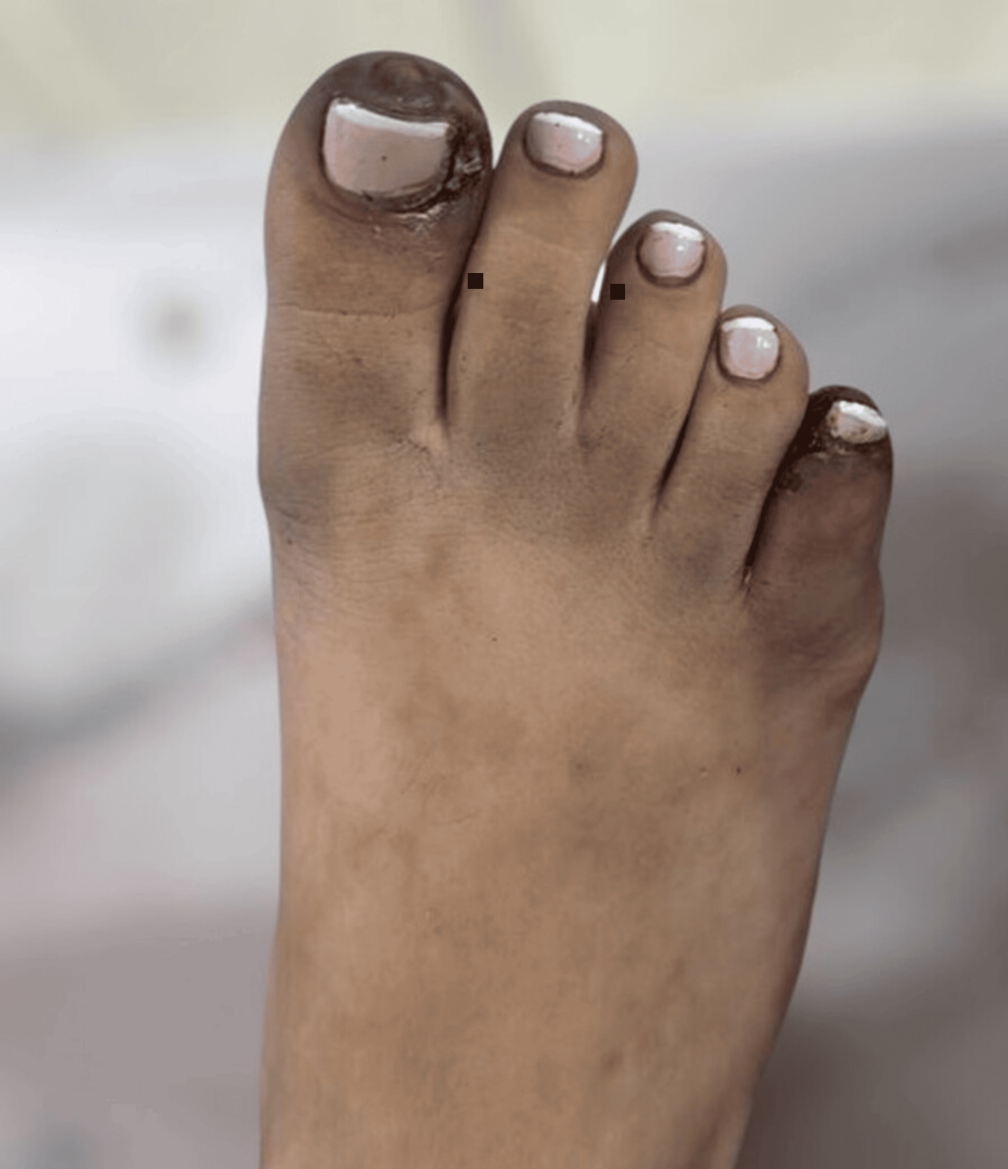
Ulcerated lesions on the hallux, fourth, and fifth toes of the right foot.

On admission, physical examination revealed normal vital signs with normal cardiopulmonary auscultation, a soft, non-tender abdomen without organomegaly, and no peripheral lymphadenopathy, dermatological abnormalities, joint swelling, or neurological deficits. Examination of the right lower limb revealed cyanosis of all toes, predominantly affecting the hallux and fifth toe (Figure 1), delayed distal capillary refill, hyperalgesia, and absence of anterior and posterior tibial pulses; the popliteal pulse was diminished. The left lower limb exhibited preserved pulses, except for a decreased anterior tibial pulse.

Laboratory work-up (Table 1) demonstrated elevated antiphospholipid antibody levels (IgG and IgM anticardiolipin antibodies, IgG and IgM β2-glycoprotein I antibodies, and elevated lupus anticoagulant), elevated antinuclear antibodies (ANA), a prolonged activated partial thromboplastin time, hypocomplementemia with decreased C3 and C4 levels, a positive direct Coombs test, a reactive Venereal Disease Research Laboratory (VDRL) test, and elevated antithrombin III. Other etiologies of genetic thrombophilia, such as protein S and antithrombin III deficiencies and factor V Leiden mutation, were ruled out.

**Table 1:** Laboratory results. The laboratory results were consistent with systemic autoimmunity and acquired thrombophilia. Key findings included elevated antiphospholipid antibody levels, positive antinuclear antibodies (ANA), hypocomplementemia, prolonged activated partial thromboplastin time (aPTT), a positive direct Coombs test, and a reactive Venereal Disease Research Laboratory (VDRL) test. RNP: ribonucleoprotein.

Detection index	Patient result	Reference range
Complete blood count	Normal ranges	
IgG anticardiolipin antibodies	67.3	<12
IgM anticardiolipin antibodies	15.2	<12
IgG β2-glycoprotein I antibodies	34.2	<20
IgM β2-glycoprotein I antibodies	10.8	<20
Lupus anticoagulant	69.6	0–12
Antinuclear antibodies (ANA)	1:320	<1:80
Activated partial thromboplastin time (aPTT)	38.7	29.9
C3 complement	76	90–180
C4 complement	4.1	10–40
Direct Coombs test	+++	Negative
VDRL	Reactive (1:4)	Non-reactive
Antithrombin III	147.9	83–116
Protein S	92	80–130
Factor V Leiden	Negative	Negative
Anti-RNP antibodies	3.7	0–10
Anti-Sm antibodies	3.6	0–10
Anti-Ro/SSA antibodies	4.6	0–10
Anti-La/SSB antibodies	4.6	0–10

**Figure 2:**
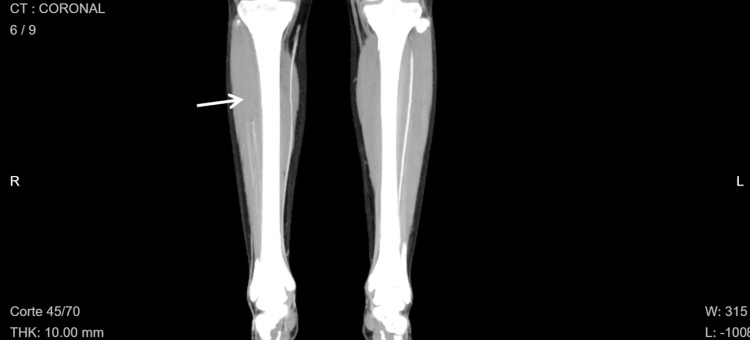
Coronal computed tomography image of the lower limbs. The arrow indicates decreased attenuation of the right anterior tibial artery, consistent with arterial occlusion.

**Figure 3:**
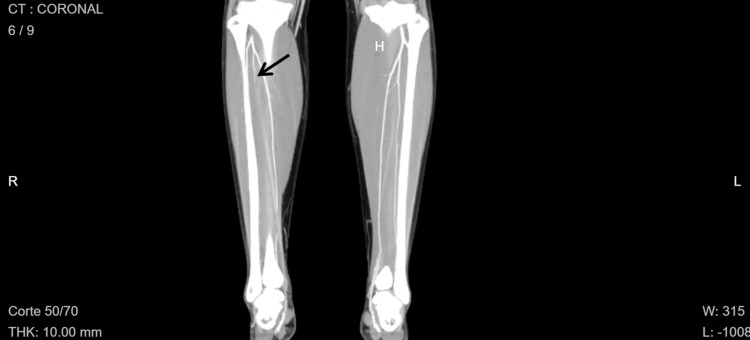
Coronal computed tomography image of the lower limbs. The arrow indicates occlusion of the proximal segment of the right peroneal artery.

Computed tomography angiography of the extremities confirmed arterial occlusion of the right lower extremity, showing occlusion at the junction of the proximal-middle third of the right anterior tibial and peroneal trunks with minimal distal recanalization of flow in the dorsalis pedis artery (Figures 2, 3). No other segments of the occlusion were observed. A transthoracic echocardiogram showed normal cardiac anatomy and function, normal pulmonary artery pressures, no intracardiac shunts, and no intracavitary thrombi.

Based on the clinical presentation, a differential diagnosis was considered, including vasculitis of large vessels, congenital heart disease with embolic events, popliteal artery entrapment syndrome, trauma, or iatrogenic arterial injury, and inherited thrombophilia. These conditions were excluded based on clinical history, imaging, echocardiographic findings, and laboratory results. Based on these findings, the patient was diagnosed with antiphospholipid syndrome secondary to systemic lupus erythematosus with thrombotic manifestations.

The patient was initially treated with three pulses of methylprednisolone 500 mg (10 mg/kg) intravenously (IV), followed by a transition to oral prednisone 50 mg daily (1 mg/kg). Anticoagulation therapy consisted of enoxaparin 60 mg administered subcutaneously (SC) every 12 hours (1.15 mg/kg). Antiplatelet therapy included aspirin 100 mg orally once daily (1.9 mg/kg). Clindamycin 500 mg IV every 8 hours (9.6 mg/kg) was administered for seven days as prophylaxis against secondary infection. Immunosuppressive therapy included chloroquine 250 mg (150 mg base) orally once daily (4.8 mg/kg). Additionally, she received amlodipine 5 mg orally once daily (0.1 mg/kg) for vascular support and atorvastatin 20 mg orally once daily (0.38 mg/kg) to improve perfusion.

The patient was managed by a multidisciplinary team: pediatric rheumatology guided diagnosis and immunosuppressive treatment; hematology directed anticoagulation and thrombophilia assessment; vascular surgery monitored limb viability and ruled out surgical intervention; infectious diseases managed antibiotic prophylaxis; and orthopedics contributed by excluding musculoskeletal causes.

This multimodal therapeutic approach aimed to control lupus disease activity, reduce thrombotic risk, and improve endothelial function and tissue perfusion. Clinical improvement was evidenced by complete resolution of pain and hyperalgesia, recovery of palpable distal pulses, normalization of capillary refill, and healing of necrotic skin lesions. She avoided surgery and was discharged on outpatient therapy with continued immunosuppressive, anticoagulant, and antiplatelet treatment. At the three-month follow-up, the patient remained clinically stable, with no recurrence of thrombotic events, complete healing of skin lesions, and ongoing maintenance of immunosuppressive, anticoagulant, and antiplatelet therapy.

Written informed consent was obtained from the patient's legal guardian for publication of this case report and the accompanying images, with all identifying information removed to protect patient privacy. Institutional review board approval was not required for single-patient case reports in our institution.

## Discussion

Few childhood cases of APS associated with SLE have been reported. In the pediatric age group, primary APS is the most frequent form of presentation, followed by APS secondary to SLE, which is estimated to occur in 39% of the cases [5]. The primary clinical manifestation is deep vein thrombosis, whereas arterial thrombosis is rare. Cerebral circulation is most affected in patients with APS who develop arterial thrombosis, while peripheral arterial involvement is even rarer [6]. According to Morad et al. [7], women present mostly with venous thrombosis at a younger age, while men with arterial events later in life suffer more recurrent events. In general, APS is more commonly diagnosed in middle-aged adults. 

Wurster et al. [8] reported the case of a six-year-old girl diagnosed with APS secondary to SLE who presented with thrombosis of the right popliteal artery and cyanotic discoloration of the second to fifth toes of the right foot. She also experienced abdominal pain, fever, and arthralgia. Her most relevant laboratory results showed pancytopenia, hypocomplementemia, and the presence of ANA antibodies, anticardiolipin, and lupus anticoagulant, an antibody which, despite its name, is associated with an increased risk of thrombosis. In contrast, our patient only presented with signs of peripheral arterial occlusion as the main complaint. Non-thrombotic hematologic manifestations, such as hemolytic anemia (in the context of a positive direct Coombs test), thrombocytopenia, leukopenia, or Evans syndrome, which can be found in 38% of children with APS, were absent [9]. She also did not present with arthralgia, headaches, fever, or weight loss, which are non-specific but common symptoms of juvenile SLE [10]. 

The patient's clinical presentation, characterized by a severe arterial thrombotic event without the typical systemic symptoms of juvenile SLE (such as arthralgia, headaches, or fever), was a significant diagnostic challenge. The silent nature of the underlying SLE in this case required a high index of suspicion for autoimmune conditions like SLE and APS, even in the absence of a full set of diagnostic criteria. Despite this atypical presentation, our patient did have a positive VDRL test result. False-positive results on nontreponemal tests can be found in up to 20% of patients with SLE and are closely associated with the presence of APS [11]. A retrospective study of 58 pediatric patients revealed that those with secondary APS more frequently had hypocomplementemia and ANA antibodies [12]. These findings were also observed in our patient. In addition, the presence of antiphospholipid antibodies and the arterial occlusion detected on imaging were key to establishing the diagnosis and helped exclude other potential causes, such as hereditary thrombophilia.

A thorough diagnostic work-up was crucial to differentiate APS from other conditions presenting with similar symptoms. For instance, a transthoracic echocardiogram was performed to rule out a cardiac source for the thrombus, such as intracavitary thrombi or intracardiac shunts, which could cause embolic events. The patient's history, without any recent trauma or surgical interventions, helped exclude trauma or iatrogenic arterial injury as the cause of the occlusion. Additionally, the normal cardiac anatomy and function on the echocardiogram and the absence of a history of repeated vascular compression ruled out popliteal artery entrapment syndrome and other congenital heart diseases. The exclusion of genetic thrombophilia, such as protein S and antithrombin III deficiencies and factor V Leiden mutation, further supported the diagnosis of acquired thrombophilia associated with autoimmunity. Ultimately, the combination of clinical presentation, specific serological markers (elevated aPL and ANA), and the characteristic findings on computed tomography angiography strongly supported the diagnosis of APS secondary to SLE.

The development of thrombotic events requires a "first hit,” given by the presence of antiphospholipid antibodies, and the presence of a "second hit," corresponding to inflammatory triggers [13]. These include atherosclerosis, smoking, hypertension, and contraception in adults. However, these prothrombotic factors are unlikely in children, with infections being an important trigger [2]. Although we did not identify "second hit" factors in our patient, the symptoms of acute peripheral arterial occlusion, such as intermittent claudication-type pain, were likely caused by other mechanisms, including endothelial cell proliferation, intimal hyperplasia, endothelial dysfunction, platelet activation, and complement consumption [13].

The clinical presentation of APS in children also differs from that of adults. Pediatric APS is a rare condition where diagnosis can be challenging if adult classification criteria are applied, as non-thrombotic manifestations such as thrombocytopenia and hemolytic anemia may precede thrombotic events [1]. Furthermore, cerebrovascular thrombosis is more frequent in pediatric patients (32%) compared to adults (16-21%), highlighting a different pattern of disease [1]. Another key distinction is the progression from primary APS to SLE, which is four times more common in children (21%) than in adults (0.6%), underscoring a more aggressive course of the disease in the younger population [1].

Currently, there are classification criteria for APS and SLE in adults; however, there are no validated criteria in the pediatric population [2,14]. To address this gap, the Single Hub and Access point for Pediatric Rheumatology in Europe (SHARE) initiative published in 2017 evidence-based recommendations for the diagnosis and management of pediatric APS and its association with SLE [15]. These guidelines recommend a diagnostic approach that includes antiphospholipid antibody testing, complement levels, autoantibodies, and imaging studies, such as echocardiography and angiography, to evaluate cardiovascular and thrombotic involvement. For treatment, the guidelines suggest a combination of corticosteroids, immunosuppressive agents, and anticoagulation, with antiplatelet therapy considered in selected cases [15].

In our case, management aligned with these core principles: the patient received corticosteroids, anticoagulation (enoxaparin), antiplatelet therapy (aspirin), and the immunomodulator chloroquine as part of lupus-specific therapy. Additionally, she was treated with amlodipine and atorvastatin to support vascular function and perfusion, interventions not specifically addressed by the guidelines but deemed appropriate based on clinical judgment. Despite prompt treatment, the case illustrates how the clinical and laboratory heterogeneity seen in pediatric APS and SLE can delay diagnosis and complicate therapeutic decisions [2,16].

Catastrophic antiphospholipid syndrome (CAPS) is the most severe and acutely life-threatening variant of APS, characterized by rapid multiorgan involvement and widespread small vessel thrombosis within days, along with the presence of antiphospholipid antibodies. In pediatric cases, CAPS has been managed with immediate combination therapy, including short-term anticoagulation, corticosteroids, and plasma exchange, with or without intravenous immunoglobulin (IVIG), as recommended by the European SHARE initiative [15]. In neonates with APS, routine anticoagulation is generally not indicated [5]. Furthermore, the treatment in the pediatric population can be particularly challenging, especially for APS secondary to SLE, which has a worse prognosis and higher mortality compared with adult patients [4]. The mortality rate in children is reported to be around 7%, with the primary cause of death often being thrombotic complications, whereas in adults, it is more commonly related to infections [1].

Long-term anticoagulation in pediatric APS is considered after an initial thrombotic event if antiphospholipid antibodies (aPLs) persist, while it may not be necessary if aPLs disappear. In cases of recurrent thrombosis despite standard anticoagulation (international normalized ratio (INR) 2.0-3.0), a higher target INR (3.0-4.0) or prolonged low-molecular-weight heparin therapy can be considered. However, long-term anticoagulation carries significant bleeding risks, especially during physical activities. Similarly, aspirin prophylaxis is generally not recommended in asymptomatic aPL-positive children, as the risk of bleeding during play and sports may outweigh its potential benefits. Therefore, decisions regarding long-term anticoagulation should be individualized, weighing the risk of thrombosis against potential complications [5].

In our case, the patient responded well to medical treatment and, to date, has not experienced a thrombotic recurrence three months post-treatment. However, this favorable short-term outcome must be interpreted with caution within the context of existing literature. Studies on pediatric APS and SLE show a high rate of recurrence for thrombotic events, with recurrence rates as high as 19% over a six-year follow-up period [1]. Furthermore, the long-term prognosis of APS secondary to SLE in children is often guarded due to the risk of organ damage and the potential for future thrombotic events [1]. Therefore, despite a positive initial response to treatment, close monitoring and adherence to a long-term anticoagulation strategy remain critical to preventing future complications [5].

In patients with APS who have recently experienced a thrombotic event, elective surgery should be delayed by at least three months due to the potential risk of rethrombosis or progression to catastrophic antiphospholipid syndrome [17]. In our case, the patient responded well to medical treatment, and surgical intervention was not necessary.

In pediatric populations, it is important to recognize that antiphospholipid antibodies (aPLs) should be screened in appropriate clinical settings, such as thrombosis, multiorgan dysfunction, or concomitant systemic autoimmune diseases [18]. Improving awareness and early recognition of signs and symptoms can lead to earlier diagnosis and increase the chances of recovery.

## Conclusions

Clinicians should maintain a high index of suspicion for antiphospholipid syndrome (APS) in young patients presenting with thrombotic events and consider autoimmune screening when appropriate. Early recognition, prompt diagnosis, and a comprehensive treatment approach, delivered with coordinated input from rheumatology, hematology, vascular surgery, and other relevant specialties, can prevent irreversible complications such as limb loss.

Our case illustrates that even in the absence of classic systemic features or identifiable prothrombotic triggers, pediatric APS secondary to systemic lupus erythematosus (SLE) can present with isolated peripheral arterial occlusion. In such scenarios, strict adherence to guideline-based diagnostic work-up and timely initiation of individualized multimodal therapy can achieve full recovery and avoid surgical intervention. Further research is needed to refine pediatric-specific diagnostic criteria and to optimize management strategies for APS associated with SLE.
